# Survival from melanoma of the skin in England and Wales up to 2001

**DOI:** 10.1038/sj.bjc.6604585

**Published:** 2008-09-23

**Authors:** B Rachet, M J Quinn, N Cooper, M P Coleman

**Affiliations:** 1 Cancer Research UK Cancer Survival Group, Non-Communicable Disease Epidemiology Unit, Department of Epidemiology and Population Health, London School of Hygiene and Tropical Medicine, Keppel Street, London WC1E 7HT, UK; 2 Social and Health Analysis and Reporting Division, Office for National Statistics (Room FG/114), 1 Myddelton Street, London EC1R 1UW, UK

Melanoma is unusual among tumours of adults in that it is generally defined by its morphology, not its anatomic location. Melanoma arises from melanocytes that are found mostly in the skin, but also in the bowel and the uveal and genital tracts. Only melanoma of the skin is considered here, including the very rare melanomas of the scrotal skin, defined both by anatomic location (ICD-9 1877, ICD-10 C632) and morphology.

Melanoma of the skin has been increasing rapidly in the United Kingdom since the 1960s, as in many other countries (Coleman *et al*, 1993). Only prostate cancer rose more rapidly during the 1990s (Quinn *et al*, 2001). Almost 7000 cases are now diagnosed in England and Wales each year, almost double again the figure for 1990 (3700), itself double the number of cases in 1970. Melanoma now ranks as the sixth most frequent malignancy in women, accounting for 3% of tumours. The annual incidence rate in women (15 per 100 000) is higher than in men (12), and the risk is 2–3 times higher among the most affluent groups: both features are unusual for a malignancy. The increase in risk in England and Wales affects all ages, both sexes and all socioeconomic groups, although the rise has been more marked in the elderly, in women and in the most affluent (data not shown).

Increases in mortality during the 1970s and 1980s were less marked than for incidence, and mortality trends slowed further during the 1990s, with a rise of only 8% in men and an actual fall of 12% in women. Stable or falling mortality alongside rapidly increasing incidence suggests substantial gains in survival.

We report here the survival patterns for over 55 000 adults who were diagnosed with malignant melanoma of the skin in England and Wales during 1986–1999 and were followed up to 31 December 2001. They represent 92% of patients eligible for inclusion in the analyses. Some 2% of patients were excluded because their vital status was not known when the data were extracted for analysis on 2 November 2002, a further 3% because their duration of survival was zero or unknown, and another 3% because the melanoma was not their first invasive primary malignancy (data not shown).

The skin of the leg and hip is still the most common location for a melanoma, but the distribution has shifted towards the trunk over the last 30 years. The leg and hip accounted for 45% of all melanomas in the early 1970s, but this figure has fallen steadily, reaching 32% by the late 1990s. The proportion arising on the skin of the trunk excluding the scrotum rose from 18 to 25% over the same period. The skin of the face, head and neck has accounted for a steady 15–16% throughout these three decades.

Virtually all the tumours were assigned to one of the morphology codes for malignant melanoma, and only 2% had a nonspecific morphology code. As melanomas of the skin are primarily identified by an anatomic site code (ICD-9 172, ICD-10 C43), rather than their morphology, this suggests a high standard of diagnostic accuracy. The proportion of tumours coded as superficial spreading melanoma rose from 18% to 33% during the period 1986–1999, although the proportion classified simply as malignant melanoma fell from 70 to 56%. The annual number of melanomas has trebled since the early 1970s, so even this large proportional change does not adequately reflect the increase in superficial spreading melanoma, for which the number of cases in England and Wales almost doubled from 840 to 1500 cases a year between 1991 and 1999 alone (data not shown). This increase may well be real, and although it could also reflect a change in how pathologists describe and classify melanoma, a detailed review of melanoma incidence trends suggests a remarkable constancy in the pathological definition of melanoma over time and place, at least up to the late 1980s (van der Esch *et al*, 1991).

## Survival trends

**Figure 1:**
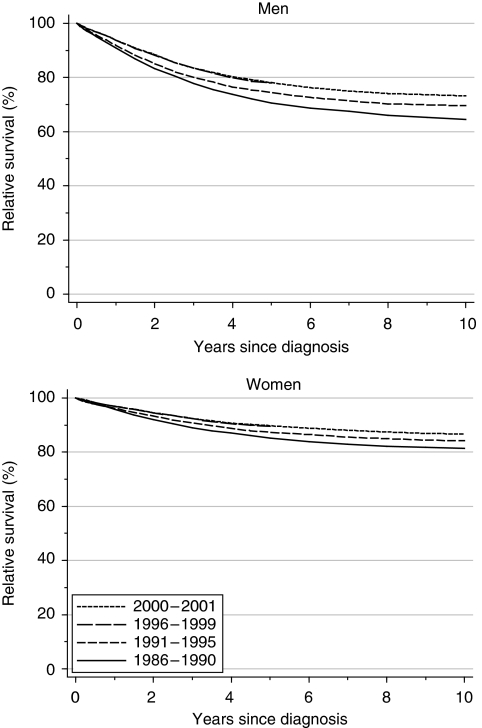
Relative survival (%) up to 10 years after diagnosis by sex and calendar period of diagnosis: England and Wales, adults (15–99 years) diagnosed during 1986–1999 and followed up to 2001. Survival estimated with cohort or complete approach (1986–1990, 1991–1995, 1996–1999) or hybrid approach (2000–2001) (see Rachet *et al*, 2008).

Survival from melanoma is high, and substantially higher in women than in men. For men diagnosed during 1986–1990, relative survival was 91% at 1 year and 71% at 5 years; the corresponding figures for women were 96 and 85%. After adjustment for deprivation, 1-year survival in men rose by approximately 2% every 5 years, reaching 94% for men diagnosed during 1996–1999 (Table 1, Figure 1). Five-year survival rose more rapidly (4% every 5 years), reaching 78% for men diagnosed 1996–1999. For women, deprivation-adjusted increases in survival were smaller, and not statistically significant.

Short-term predictions of survival, using hybrid analysis (Brenner and Rachet, 2004) of the survival probabilities observed during 2000–2001, suggests the underlying increase in survival will continue in the near future (Table 1).

## Deprivation

**Figure 2:**
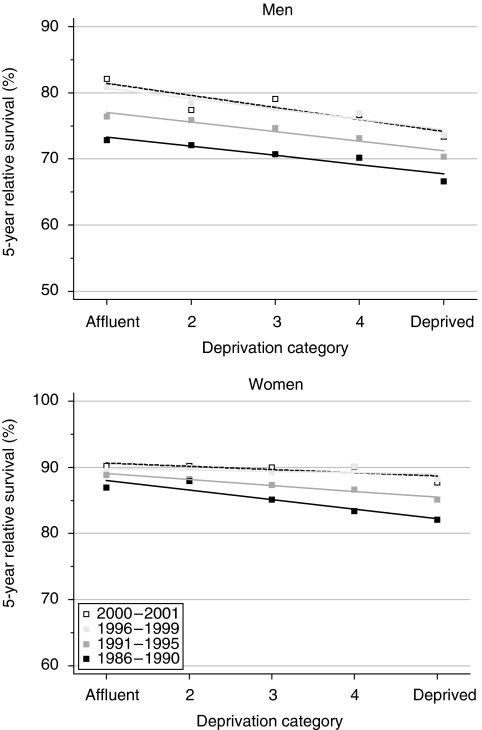
Trends in the deprivation gap in 5-year relative survival (%) by sex and calendar period of diagnosis: England and Wales, adults (15–99 years) diagnosed during 1986–1999 and followed up to 2001.

Survival up to 5 years remains 4–6% lower among men in the more deprived groups than among the more affluent, but the deprivation gap did not widen significantly during the 1990s. The deprivation gap in survival for women fell during the 1990s, and it is now only 1%. The fall in the deprivation gap in 5-year survival, 2.6% every 5 years, was statistically significant (Table 2, Figure 2).

Hybrid analysis of patients' survival experience during 2000–2001 suggests that these patterns will persist for at least 5 years, with a significant deprivation gap for men and a much smaller socioeconomic difference in survival among women (Table 2).

## Comment

Survival from melanoma of the skin in England and Wales is high, and the fact that 5-year survival is increasing so much more rapidly than 1-year survival – more so for melanoma than for any of the other malignancies examined here – suggests a real increase in survival and cure, presumably attributable to earlier diagnosis and/or improved treatment. The socioeconomic gradient in survival also appears to be stable or declining.

These encouraging trends continue those seen since the 1970s and 1980s, with more rapid increases for men than women in both short-term and long-term survival, and less marked socioeconomic inequality in survival. Thus the difference in 5-year survival between women and men fell from 21% for patients diagnosed in the early 1970s to 14% by the late 1980s (Coleman *et al*, 1999), and now to 12% by the late 1990s in these data. Similarly, the deprivation gap in survival between the most affluent and most deprived patients diagnosed during 1981–1985 was approximately 6% at 1 year and 12% at 5 years. This fell to 4 and 8%, respectively, for those diagnosed during 1986–1990, and now to 6 and 1% for patients diagnosed during 1996–1999.

Survival is higher for melanomas less than 1.5 mm in thickness at diagnosis, when radical surgery may still be curative; the prognosis for thicker tumours or disseminated disease is poor. Earlier diagnosis also contributes to socioeconomic differences in survival.

Survival patterns in Scotland suggest a possible explanation for the overall trends and the change in socioeconomic gradient in survival in England and Wales. Increasing survival in Scotland, following a public education campaign to encourage earlier diagnosis, has been ascribed to earlier diagnosis and thinner lesions (MacKie *et al*, 1992). In a population study of 3000 cases diagnosed in the west of Scotland during 1979–1993, thin melanomas were more common in the affluent, but the proportion of thin tumours increased more in deprived groups during this period (MacKie and Hole, 1996).

The fact that survival rates in Scotland for melanoma – but not for any other tumour – are significantly higher in Scotland than the European average for both men and women (Berrino *et al*, 2003), tends to reinforce the conclusion that the public awareness campaign in Scotland has been valuable in fostering earlier diagnosis, higher survival and less inequality in survival.

## Figures and Tables

**Table 1:** Trends in relative survival (%) by sex, time since diagnosis and calendar period of diagnosis: England and Wales, adults (15–99 years) diagnosed during 1986–1999 and followed up to 2001

		**Calendar period of diagnosis^a^**									
		**1986–1990**		**1991–1995**		**1996–1999**		**Average change (%) every 5 years^b^**		**Prediction^c^ for patients diagnosed during 2000–2001**	
**Time since diagnosis**		**Survival (%)**	**95% CI**	**Survival (%)**	**95% CI**	**Survival (%)**	**95% CI**	**Survival (%)**	**95% CI**	**Survival (%)**	**95% CI**
1 year	Men	**90.9**	(90.1, 91.7)	**91.9**	(91.2, 92.5)	**93.8**	(93.1, 94.4)	1.7**	(0.5, 3.0)	**93.8**	(92.9, 94.6)
	Women	**95.8**	(95.3, 96.2)	**96.2**	(95.8, 96.6)	**96.9**	(96.5, 97.3)	**0.0**	(−0.7, 0.8)	**96.9**	(96.3, 97.4)
5 years	Men	**70.6**	(69.3, 72.0)	**74.4**	(73.3, 75.5)	**77.9**	(76.5, 79.2)	4.0**	(1.6, 6.3)	**78.1**	(76.5, 79.6)
	Women	**85.2**	(84.3, 86.0)	**87.3**	(86.6, 88.0)	**89.5**	(88.6, 90.3)	**0.3**	(−1.2, 1.8)	**89.8**	(88.7, 90.7)
10 years	Men	**64.6**	(63.0, 66.1)	**69.5**	(68.1, 70.9)			**3.9**	(−1.0, 8.9)	**73.2**	(71.3, 75.0)
	Women	**81.4**	(80.4, 82.3)	**84.2**	(83.2, 85.1)			**0.9**	(−2.3, 4.2)	**86.7**	(85.4, 87.9)

CI=confidence interval.

a Survival estimated with cohort or complete approach (see Rachet *et al*, 2008).

b Mean absolute change (%) in survival every 5 years, adjusted for deprivation (see Rachet *et al*, 2008).

c Survival estimated with hybrid approach (see Rachet *et al*, 2008).

^**^P<0.01.

**Table 2:** Trends in the deprivation gap in relative survival (%) by sex, time since diagnosis and calendar period of diagnosis: England and Wales, adults (15–99 years) diagnosed during 1986–1999 and followed up to 2001

		**Calendar period of diagnosis^a^**									
		**1986–1990**		**1991–1995**		**1996–1999**		**Average change (%) every 5 years^b^**		**Prediction^c^ for patients diagnosed during 2000–2001**	
**Time since diagnosis**		**Deprivation gap (%)**	**95% CI**	**Deprivation gap (%)**	**95% CI**	**Deprivation gap (%)**	**95% CI**	**Deprivation gap (%)**	**95% CI**	**Deprivation gap (%)**	**95% CI**
1 year	Men	−3.1*	(−5.5, −0.7)	−2.3*	(−4.3, −0.3)	−3.5**	(−5.2, −1.7)	**−0.3**	(−1.9, 1.2)	−4.2**	(−6.7, −1.7)
	Women	−2.8**	(−4.1, −1.4)	−1.7**	(−2.9, −0.5)	−1.3*	(−2.5, −0.1)	**0.8**	(−0.2, 1.7)	**−1.4**	(−3.1, 0.2)
5 years	Men	−5.6**	(−9.5, −1.6)	−5.8**	(−9.1, −2.5)	−6.2**	(−10.2, −2.3)	**−0.4**	(−3.3, 2.6)	−7.3**	(−11.8, −2.7)
	Women	−5.8**	(−8.3, −3.2)	−3.6**	(−5.7, −1.4)	**−0.9**	(−3.4, 1.6)	2.6**	(0.7, 4.4)	**−1.9**	(−5.0, 1.1)
10 years	Men	−6.0**	(−10.4, −1.6)	−4.8*	(−8.9, −0.6)			**1.2**	(−4.8, 7.3)	**−4.9**	(−10.3, 0.5)
	Women	−7.8**	(−10.7, −4.9)	−5.2**	(−8.0, −2.3)			**2.7**	(−1.4, 6.8)	**−3.2**	(−6.9, 0.6)

CI=confidence interval.

a Survival estimated with cohort or complete approach (see Rachet *et al*, 2008).

b Mean absolute change (%) in the deprivation gap in survival every 5 years, adjusted for the underlying trend in survival (see Rachet *et al*, 2008).

c Survival estimated with hybrid approach (see Rachet *et al*, 2008).

^*^P<0.05; ^**^P<0.01.
